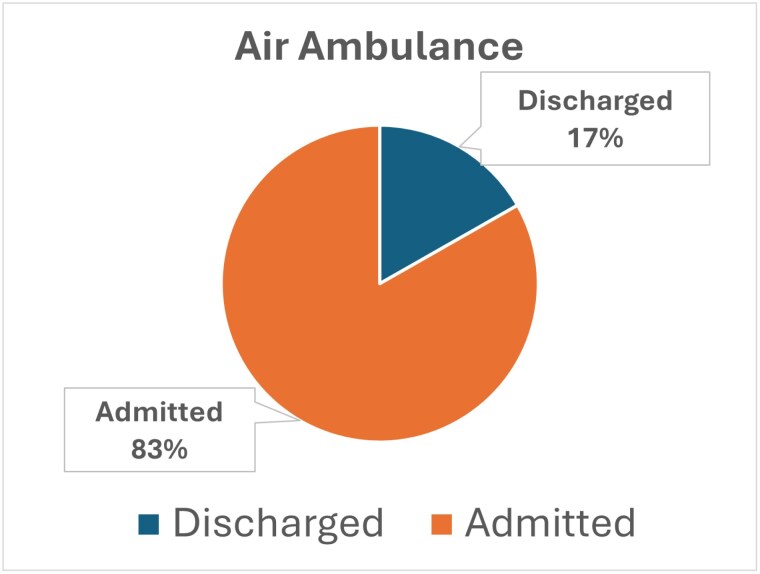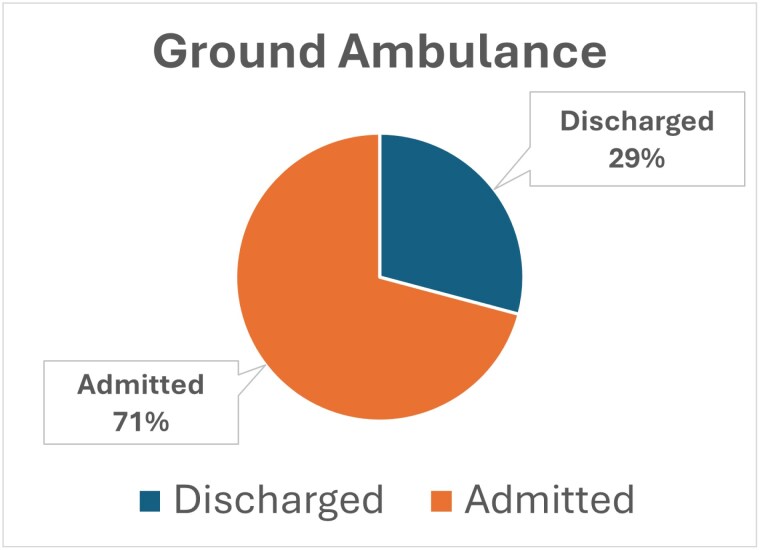# 614 Burn Center Transfers by Ambulance: Economic Opportunity and Consequences of Burn Center Destination Triage

**DOI:** 10.1093/jbcr/iraf019.243

**Published:** 2025-04-01

**Authors:** Randy Kearns, Anastasiya Ivanko, Jonathan Schoen, Herbert Phelan, William Hickerson, Jeffrey Carter

**Affiliations:** University of New Orleans; Burn Center at University Medical Center; Louisiana State University Health Sciences Center; Louisiana State University Burn Center; Louisiana State University Health Sciences Center; Louisiana State University Burn Center

## Abstract

**Introduction:**

Of the 139mil emergency department visits in the United States in 2022, approximately 700k (0.50%) were for burn injuries requiring emergent evaluation and medical care. While burn injuries remain uncommon, this infrequency often leaves local clinicians without sufficient experience in dealing with them. This lack of knowledge can contribute to under- and over-triage.

**Methods:**

Relying on the American Burn Association, Burn Care Quality Platform Data Warehouse, the query included patients with an initial admission over three years (2020-2022). We used industry-common reimbursements for the appropriate means of transport. We included two transport modes (ground and helicopter ambulance). The analysis included the financial impact and the opportunity costs associated with over-triage scenarios.

**Results:**

The base population over three years, adjusted for age, burn etiology, and no trauma diagnosis, was 46,328 cases. Transfers were defined as all patients who do not have a “Not Referred/Transferred From Another Facility” value for Patient Transfer. Of the 43,328 cases, 18,305 were transported by ground ambulance, with 5,337 (29.2%) discharged in one or fewer days. Of the 43,328 cases, 2571 were transferred by helicopter ambulance, and 431 (16.8%) were discharged in one or fewer days. Relying on transportation costs derived from the CMS Ambulance Fee Schedule Public Use Files, we identified ground ambulance costs of $1551 (per transport) and helicopter ambulance costs of $9,090 (per transport).

Assuming those discharged in less than one day could have either been consulted by telehealth, treated and released at a local hospital with home care instructions, or treated and released at a local hospital with a personal vehicle transport to a burn center clinic, the potential savings for the three years was $8,277,687 (ground ambulance) and $3,917,790 (helicopter ambulance), totaling $12,145,477.

If we assume 50% of these patients could have benefited from a less than one-day stay at a burn center, the FO ($12,145,477 – CO ($6,072,738) leaves an opportunity cost of $6,072,738.

**Conclusions:**

There are situations where patients should be transferred to a burn center but are (for whatever reason) treated at a community hospital (non-burn center). These results also remind us that there are patients transferred by helicopter or ground ambulance who could most likely benefit from less expensive options. The infrequency of burn injury occurrence often leaves clinicians without sufficient experience in dealing with burn injuries. This lack of experience can contribute to under and over-triage. Just as there is potential harm for those who should have been seen at a burn center, there are tangible savings when clinicians make accurate triage decisions.

**Applicability of Research to Practice:**

Improving triage skills for community hospital staff and leveraging telehealth technologies may produce significant savings by reducing the number of over triaged patients.

**Funding for the Study:**

N/A